# ZBP-89 and Sp1 contribute to Bak expression in hepatocellular carcinoma cells

**DOI:** 10.1186/s12885-018-4349-y

**Published:** 2018-04-13

**Authors:** Xia Kong, Pin Xu, Wei-Jie Cai, Huai-Gao Wang, Bin-Bin Li, Guo-Liang Huang, Zhi-Wei He, George Chen, Cai-Guo Ye

**Affiliations:** 1 China-America Cancer Research Institute, Guangdong Medical University, Guangzhou, Guangdong Province China; 2 Guangdong Provincial Key Laboratory of Medical Molecular Diagnostics, Guangdong Medical University, Guangzhou, Guangdong Province China; 3 Department of Surgery, The Chinese University of Hong Kong, Hong Kong, SAR China

**Keywords:** Sp1, Sp3, ZBP-89, Bak, Hepatocellular carcinoma

## Abstract

**Background:**

Kruppel family member zinc binding protein 89 (ZBP-89), also known as ZNF148, regulates Bak expression via binding to GC-rich promoter domain. It is not clear if other GC-rich binding factors, such as Sp family members, can interact with ZBPp-89 on Bak expression. This study aims to elucidate the mechanism of Bak expression regulation by ZBP-89 and Sp proteins, based on in vitro experiment and The Cancer Genome Atlas (TCGA) hepatocellular carcinoma (HCC) data cohort.

**Methods:**

We downloaded TCGA hepatocellular carcinoma (HCC) cohort data to analysis the association of Bak transcription level with ZBP-89 and Sp proteins transcription level. HCC cell lines and liver immortal non-tumour cell lines were used for mechanism study, including western blotting analysis, expression vector mediated gene expression and siRNA interference.

**Results:**

Results showed that cancer tissues have higher Bak transcription level compared with adjacent non-cancer tissues. Bak transcription level was correlated with Sp1 and Sp3 expression level, while no correlation was found in ZBP-89 and Bak, neither Sp2 nor Sp4. Mithramycin A (MMA) induced Bak expression in a dose-dependent manner. Western blotting results showed Sp1 overexpression increased Bak expression both in liver immortal non-tumour cells and HCC cells. Interference Sp1 expression could inhibit Bak expression alone. ZBP-89 siRNA suppressed Bak expression even in the presence of MMA treatment and S1 overexpression. Additionally, Bak and Sp1 level were associated with HCC patient survival.

**Conclusions:**

Bak expression required ZBP-89 and Sp1 cooperative regulation simultaneously.

**Electronic supplementary material:**

The online version of this article (10.1186/s12885-018-4349-y) contains supplementary material, which is available to authorized users.

## Background

The Specificity protein (Sp) family members are GC boxes and CACCC boxes binding transcription factors, which are reported to participate in development, differentiation and tumorigenesis [1, 2]. Sp1 is also a C2H2-type zinc fingers Krüppel-like factor, and it is the first identified transcription factor [3]. Numerous studies showed that Sp1 not only acted as a basal transcription factor, but also contributed to the regulation of many vital cellular genes [4, 5]. Overexpressed Sp1 differentially regulated a large number of genes which promoted cancer development, angiogenesis and metastasis [6]. Sp3 is very similar to Sp1 both in structure and regulatory function. Sp3 and Sp1 showed equal affinity to GC box, indicating the potential competition in genes expression regulation [7]. Reversely, Sp3 mediated transcriptional repression when it binded to GC-box, because Sp3 contained a suppressive domain [8]. Additionally, Sp3 lacked the multi-merization domain, and thus Sp3 did not have the ability to super-activate promoters by bridging multiple Sp binding sites [9].

ZBP-89, also known as ZNF148, is a Krüppel-type zinc-finger transcription factor that binds to GC-rich sequences to activate or suppress gene transcription [10]. ZBP-89 is also a member of the C2H2 zinc finger family subclass, as well as Sp family members. It is known that ZBP-89 participates in many genes transcription, such as Vimentin, Gastrin, p16, and Ornithine decarboxylase et al. These biological procedures are involved in cell growth, cell cycles, metabolism and T cell immunity. Study showed ZBP-89 competed with Sp1 for binding to induce Gastrin expression when ZBP-89 functions as a repressor [11]. Interestingly, because Sp1 itself did not bind to the promoter element, ZBP-89 interacted with Sp1 to suppress Vimentin expression in vitro [12]. In another case, ZBP-89 binded to the basal promoter of Pdcd4, also interacted with Sp family members to induce Pdcd4 protein expression [13].

Bak gene belongs to a large Bcl2 family, and acts as a pro-apoptosis protein to facilitate cellular apoptosis. It penetrates and makes pores in the mitochondrial membrane through forming heterodimer with Bax [14, 15]. The penetration releases cyto C into cytoplasm, and classic apoptosis signal pathway in the end [16, 17]. Currently, several cis-elements and associated factors are required to regulate expression of the bak gene. It was reported that Sp3 competed with Sp1 to transcriptional binding sites to increase Bak expression [18]. Up-regulation of Bak by butyrate in the colon was associated with increased Sp3 binding which did not contain a multimerization domain [18]. Instead, it contains an inhibitory domain located at N-terminal to its DNA-binding domain, which mediates transcriptional repression in some instances. Mithramycin A (MMA) is a GC-rich DNA binding chemical, which is also regarded as potent Sp family members inhibitor [19]. In the Bak promoter analysis, there are many GC-rich sequence domain, indicating the possibility regulation by MMA,Sp family and ZBP-89 transcription factor. Previously, we reported that ZBP-89 bound to Bak gene promoter, and epigenetically regulated its transcription companied with histone deacetylase 3 and DNA methyltransferase 1 [20]. Therefore, we speculate that ZBP-89 and Sp1/Sp3 are involved in hepatocellular carcinoma (HCC) and Bak induction. This study focuses on the elucidation of transcription factor Sp1/Sp3 and ZBP-89 involved Bak expression.

## Methods

### Patient cohort and mRNA quantification data

HCC patient cohort information and data of mRNA quantification data were downloaded from The Cancer Genome Atlas (TCGA) database (https://portal.gdc.cancer.gov/). Previously, we published the work how we got and analyzed the patient cohort and data [21]. Totally 377 HCC patients with detailed mRNA expression level were collected. Additionally, 41 patients have non-tumor liver sample message which indicated 41 paired of tumor and non-tumor data were also included. Due to the missing follow-up survival data of some patients, we excluded those patients in the analysis of protein associated survival. Actually, 318 patients were collected for Kaplan–Meier survival analysis. Referring to published paper [21], cut-off point for gene grouping was set up and for survival analysis.

### Chemicals and drugs

Antibodies against ZBP-89 (#sc-398148), Sp1 (#sc-420) and Sp3 (sc-28305) were obtained from Santa Cruz Biotechnology (Santa Cruz, CA). Antibodies against Bak (#12105) and Actin (#3700) were obtained from Cell Signaling Technology (Beverly, MA). Ad-ZBP-89 viral vector was a generous gift from Dr. JL Merchant (University of Michigan, MI) [11]. Mithramycin A (MMA) was purchase from Cayman Inc. (#11434-1 mg). All other chemicals and reagents were purchased from Sigma-Aldrich (St. Louis, MO) unless otherwise specified.

### Cell lines

HCC cell lines, HepG2 (ATCC® HB-8065™), Hep3B (ATCC® HB-8064™), PLC/PRF/5 (ATCC® CRL-8024™) and Huh-7 (ATCC® PTA-8561™) were purchased from ATCC, and kept in our department. LO2 (also called L-02, Accession number: CVCL_6926, # GNHu 6 of Cellbank of China) is a spontaneously immortalized cell line of hepatocyte, which was a gift from Dr. Zhou of Tongji Medical College, Huazhong University of Science and Technology. MIHA is also an immortalized cell line of hepatocyte and is a TGF-β-sensitive hepatocyte cell. Studies have revealed that the validation of MIHA as a valuable immortal hepatocyte in research by Prof. Ming-liang He (the Chinese University of Hong Kong) [22]. MIHA cells were a generous gift from Prof. Ming-liang He. HKCI-3 and HKCI-4 were established by researchers of The Chinese University of Hong Kong affiliated Prince of Wales Hospital, and kept in the Dept. of Surgery lab [23]. HepG2 and Huh-7 were cultured with MEM medium, supplemented with 10% FBS. Hep3B, PLC/PRF/5 and HKCI-4 were cultured with high glucose DMEM, supplemented with 10% FBS.

### Western blotting

Western blotting was performed according to the previous publications with some modification [18, 19]. Briefly, cells were lysed by incubation on ice for 15 min in RIPA buffer to isolate total proteins. Proteins (30 μg)were separated on 10–15% SDS-PAGE gels and transferred to PVDF for immunoblotting. After membrane was blocked with 5% nonfat milk, the membrane was incubated with primary antibody for 2 h at room temperature, followed by incubation with a HRP-conjugated secondary antibody for 1 h at room temperature. The signal was visualized by ECL reagent kit (GE Healthcare) and analyzed by an Odyssey Scanner(Li-COR Biosciences Co.).

### Small RNA interference

The siRNA interference experiments were performed according to our previous publications [20, 24]. ZBP-89 siRNA and Sp1/Sp3 siRNA were obtained from Santa Cruz Biotechnology. SiRNA oligonucleotides were transfected using Fugene lipid (Roche) according to the manufacturer’s protocol. Before transfection, old medium was replaced with fresh medium. The transfection volume was 1/10 of culture medium, such as 200 μL for 2 mL culture medium in six-well plate. We used 1 nM siRNA for one million cells, and make up the volume to 200 μL by adding OPTI-MEM.

### Statistics

All results are expressed as Mean ± SE of at least three independent experiments. Differences between groups were examined for statistical significance using Mann-Whitney (U-test) unless indicated specially. *P*<0.05 was regarded as statistically significant difference.

## Results

### Bak transcription association with Sp1 and Sp3 expression

**Fig. 1:**
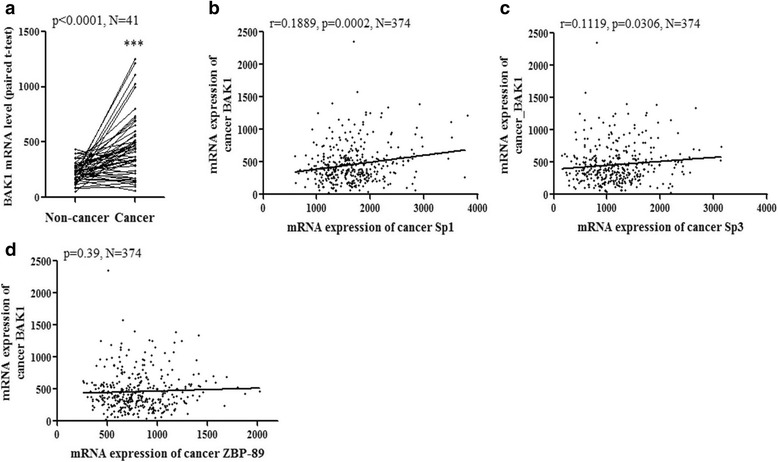
Association of Sp1/Sp3 and ZBP-89 with Bak expression in TCGA HCC patient cohort. The expression of ZBP-89, Sp1, Sp3 and Bak expression in HCC cancerous tissues and their corresponding non-cancerous tissues was plotted using expression array data. Bak mRNA level in HCC patients was significant different with corresponding non-cancer tissues (**a**, paired t-test). In the comparison of specific genes, Bak level showed significant correlation with Sp1 and Sp3 (**b** and **c**), but was negative relation with ZBP-89 expression (**d**)

In the analysis of TCGA database, the comparison of bak transcription level between 40 cases of cancer tissue and the corresponding non-cancer tissues showed increased bak transcription level (Fig. 1a, *p*< 0.001). We analyzed the correlation of Bak with ZBP-89 and Sp1–4 using SPSS correlation method. Results showed only Sp1 and Sp3 transcription level were correlated with Bak transcription level (Fig. 1b and c, r=0.1889 and *r* = 0.1119, respectively). No correlation was found in ZBP-89 and Bak (Fig. 1d), neither Sp2 nor Sp4 (data not shown). The increased transcription level of Bak was contributed to the chemotherapy drug induction or anti-cancer related ROS apoptosis induction. The clinical treatment for patients was enclosed as Additional file 1: Table S1.

### MMA increases Bak expression through blocking Sp-proteins binding activity

**Fig. 2:**
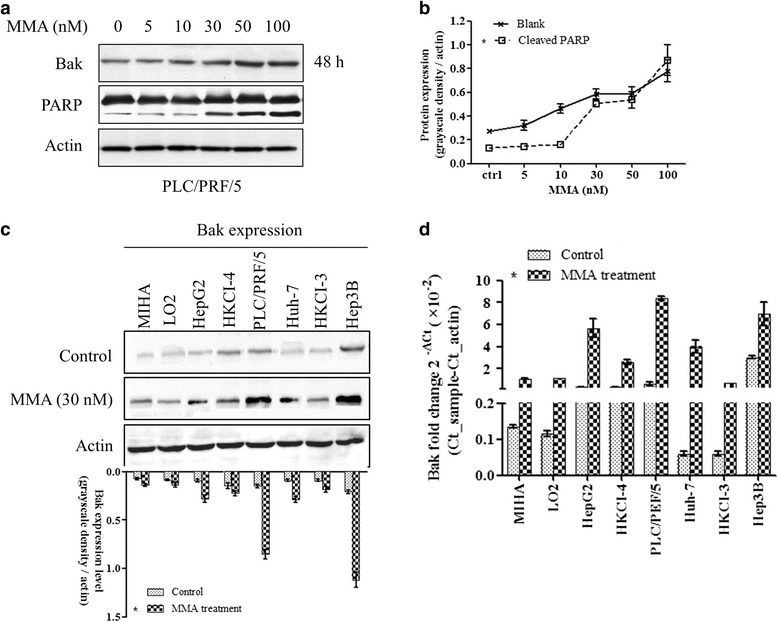
Inhibition of Sp protein function increases Bak protein expression. MMA, as a potential Sp binding inhibitor, increases Bak expression starting from the concentration of 5 nM in PLC/5 cells in 48 h incubation, and induces cleaved PARP level, which is a main mediator of apoptosis (**a**). As shown in the points and connection line figure, increased Bak expression trend is accompanied with MMA concentration from 5 nM to 100 nM, with a peak value about 5-fold increase compared with baseline (**b**, repeated three times). The inhibition rate was tested in immortal non-tumour cell lines and cancer cell lines. In the tested six HCC cell lines, MMA upregulated Bak expression (**c**, column 3 to 8). In immortal cells, MIHA and LO2, are less sensitive to MMA treatment, showing slight increasing Bak expression (C, column 1 and 2, repeated three times). MMA significantly induces Bak expression at 30 nM. In RT-PCR experiment, data showed Bak mRNA level increased significantly in HepG2, PLC/PRG/5 and Hep3B cells (D, 4 repeated wells per assay, * *p* < 0.05 compared with the corresponding control)

MMA is regarded as a potent Sp family inhibitor through displacing Sp1 binding to gene promoters. In the study, we used MMA as specific Sp proteins inhibitor. We found that increases Bak expression starting from the concentration of 5 nM in PLC/PRF/5 cells in 48 h incubation, and induces cleaved PARP level, which is a main mediator of apoptosis (Fig. 2a). Gray scale density quantification data indicated that Bak expression trend was accompanied with MMA increasing from 5 nM to 100 nM, with a peak value about 50% up-regulation compared with baseline (Fig. 2b, * *p* < 0.05 compared with control). The Bak induction rate was tested in liver immortal non-tumour cell lines and cancer cell lines. In the tested six HCC cell lines, MMA up-regulated Bak expression (C, column 3 to 8, * *p* < 0.05 compared with control), especially in PLC5/PRF/5 and Hep3B. In immortal cells, MIHA and LO2, which were less sensitive to MMA treatment, also showed increased Bak expression (C, column 1 and 2, * *p* < 0.05 compared with control). In addition, RT-PCR data showed Bak mRNA level increased significantly in HKCI-4, PLC/PRG/5 and Hep3B cells (Fig. 2d, * *p* < 0.05 compared with control). Therefore, we selected HKCI-4 and PLC/PRF/5 cancer cells as model in the subsequent study, as well as liver immortal non-tumour cells MIHA and LO2.

### Sp1 increases Bak expression but not Sp3

**Fig. 3:**
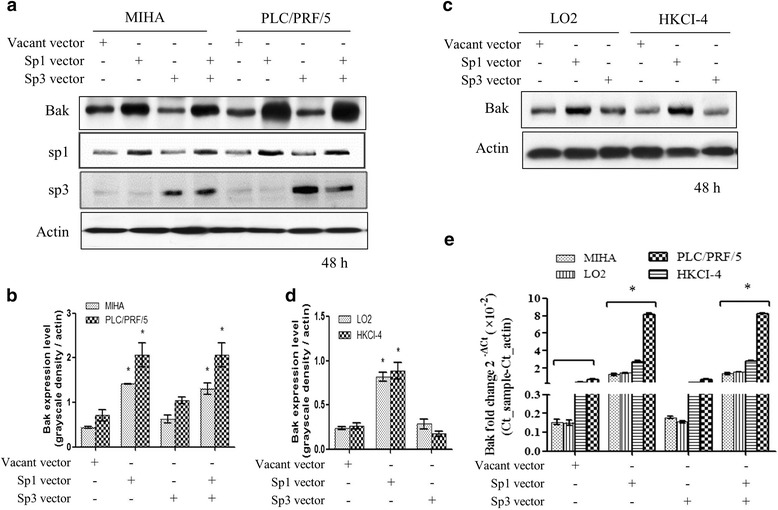
Sp1 overexpression induce Bak expression. In MIHA immortal non-tumour cells and HCC PLC/PRF/5 cells, Sp1 vector mediated upregulated expression increased Bak protein level, however, Sp3 overexpression did not affect Bak expression (**a** and **b**, * *p* < 0.05 compared with the corresponding control). In LO2 and HKCI-4 cells, Sp1 also increased Bak expression, but not Sp3 (C and D, * p < 0.05 compared with the corresponding control). RT-PCR results showed Sp1 not Sp3 induced Bak mRNA level up-regulation (E, * *p* < 0.05 compared with the corresponding control, 4-well repeated in triple experiments, data shown as Mean ± SE)

We constructed Sp1 and Sp3 expression vectors by cloning the Sp1 and Sp3 coding sequences into pcDNA3.0 respectively. When MIHA and PLC/PRF/5 cells were in density of 80% confluent, recombinant gene plasmids was transfected into cell via Fugene lipid (Roche). Western blotting results showed Sp1 overexpression increased Bak expression both in MIHA and PLC/PRF/5 cells (Fig. 3a and b, lane 1, * *p* < 0.05 compared with control), as well as in the transfection of LO2 and HKCI-4 cells (Fig. 3c and d, lane 1, * *p* < 0.05 compared with control). We did not observe that Sp3 transfection induced Bak expression. In the co-transfection of Sp1 and Sp3 vectors, Sp1 and Sp3 do not compete with binding sites. Therefore, the outcome turns to up-regulate Bak level. The results were further confirmed by RT-PCR experiment, results showed that Sp1 overexpression induced Bak up-regulation but not Sp3 (Fig. 3e, * *p* < 0.05 compared with control).

### siRNA interference Sp1 decreases Bak expression

**Fig. 4:**
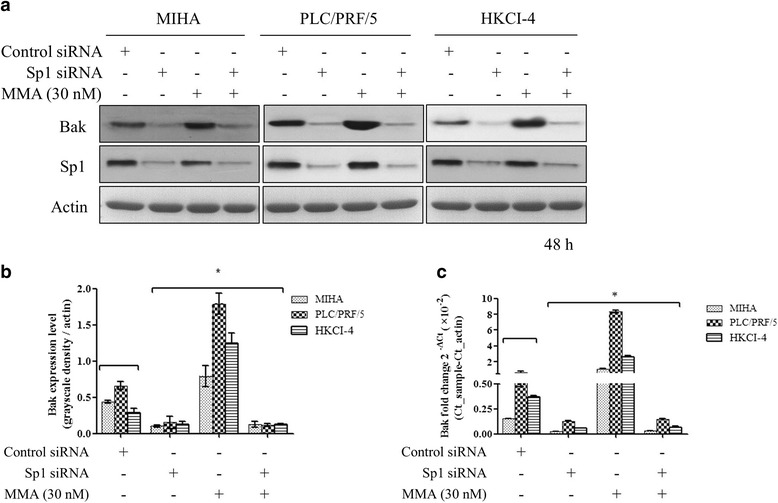
Sp1 siRNA interference decrease Bak expression. Figure showed a reduction effect on Bak expression when endogenous Sp1 was decreased by siRNA using Western blotting method (A and B, * *p* < 0.05 compared with the corresponding control). When pretreated with Sp1 expression vector, Sp1 siRNA could also decrease Bak expression level in the presence of MIHA (30 nM) in PLC/PRF/5 and HKCI-4 cells. In RT-PCR experiment of Bak mRNA level examination, data showed that Sp1 siRNA blocked Bak transcription, even in the presence of MMA treatment in MIHA, LO2, HKCI-4 and PLC/PRF/5 cells (C, * p < 0.05 compared with the corresponding control, 4-well repeated in triple experiments, data shown as Mean ± SE)

We used Sp1 siRNA nucleotide to further explore its role in Bak expression. When Sp1 siRNA was transfected in MIHA, PLC/PRF/5 and HKCI-4 cells respectively, we found that Bak expression level reduction was accompanied with the siRNA interference of Sp1 (Fig. 4a and b, lane 1, * *p* < 0.05 compared with corresponding control). Results confirmed that Bak expression level was mainly associated with Sp1 protein level and transcriptive activity. RT-PCT experiment confirmed such findings (Fig. 4c, * p < 0.05 compared with corresponding control).

### ZBP-89 cooperates with Sp1/Sp3 to facilitate Bak expression

**Fig. 5:**
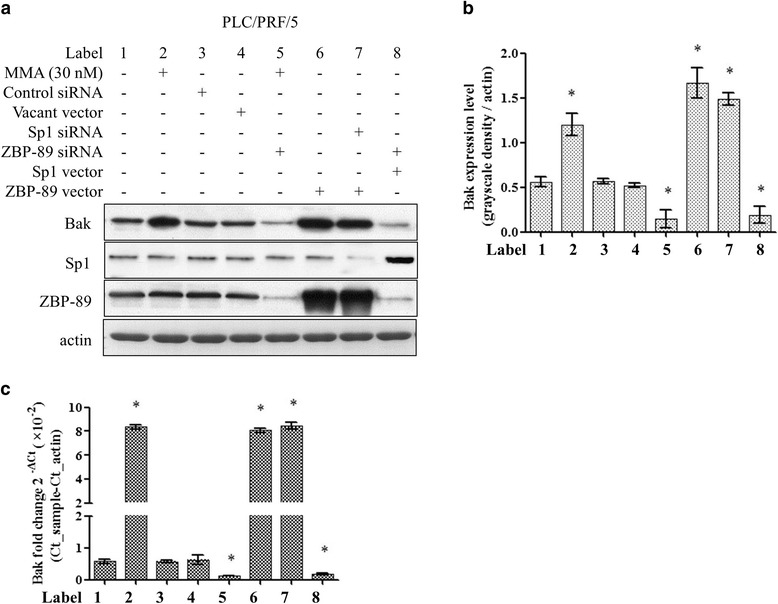
ZBP-89 and Sp1 regulated Bak expression cooperatively. Western blotting data showed that overexpressed ZBP-89 induced Bak expression, while its siRNA treatment reduced Bak expression (A and B, lane 6, * p < 0.05 compared control). Blocking ZBP-89 expression decreased Bak level even in the presence of MMA treatment, meanwhile, ZBP-89 siRNA could also abolished Sp1 overexpression mediated Bak up-regulation (**a** and **b**, lane 8, * p < 0.05 compared control). In RT-PCR experiment of Bak mRNA semi-quantification experiment, the findings in Fig. A were confirmed (C, * p < 0.05 compared control, 4-well repeated in triple experiments, data shown as Mean ± SE)

Previously, our team elucidated the epigenetic mechanism of ZBP-89 mediated Bak increasing expression via HDACs and DNMT1 activity suppression [20, 24]. In the above experiments, we have already revealed that Bak expression could be up-regulated in the presence of MMA treatment, and Sp1 vector mediated overexpression. We suspected that Bak expression might be tightly related with ZBP-89 and Sp1/Sp3 protein level. Therefore, we designed the experiment to see if ZBP-89 could interact with Sp1/Sp3 to regulate Bak expression. Western blotting data showed that siRNA blocking ZBP-89 level decreased Bak expression even with treatment of MMA (Fig. 5a and b, lane 5). Additionally, ZBP-89 siRNA interference also turned down Bak expression even though Sp1 overexpressed (Fig. 5, lane 8). In the subsequent RT-PCR analysis, data also demonstrated that ZBB-89 siRNA suppressed Bak expression in the presence of MMA treatment or Sp1 overexpression (Fig. 5c, * *p* < 0.05 compared with control). Collectively, results revealed that Bak expression required ZBP-89 and Sp1 to work simultaneously.

### Association of Bak, Sp1/Sp3 and ZBP-89 in patient survival

**Fig. 6:**
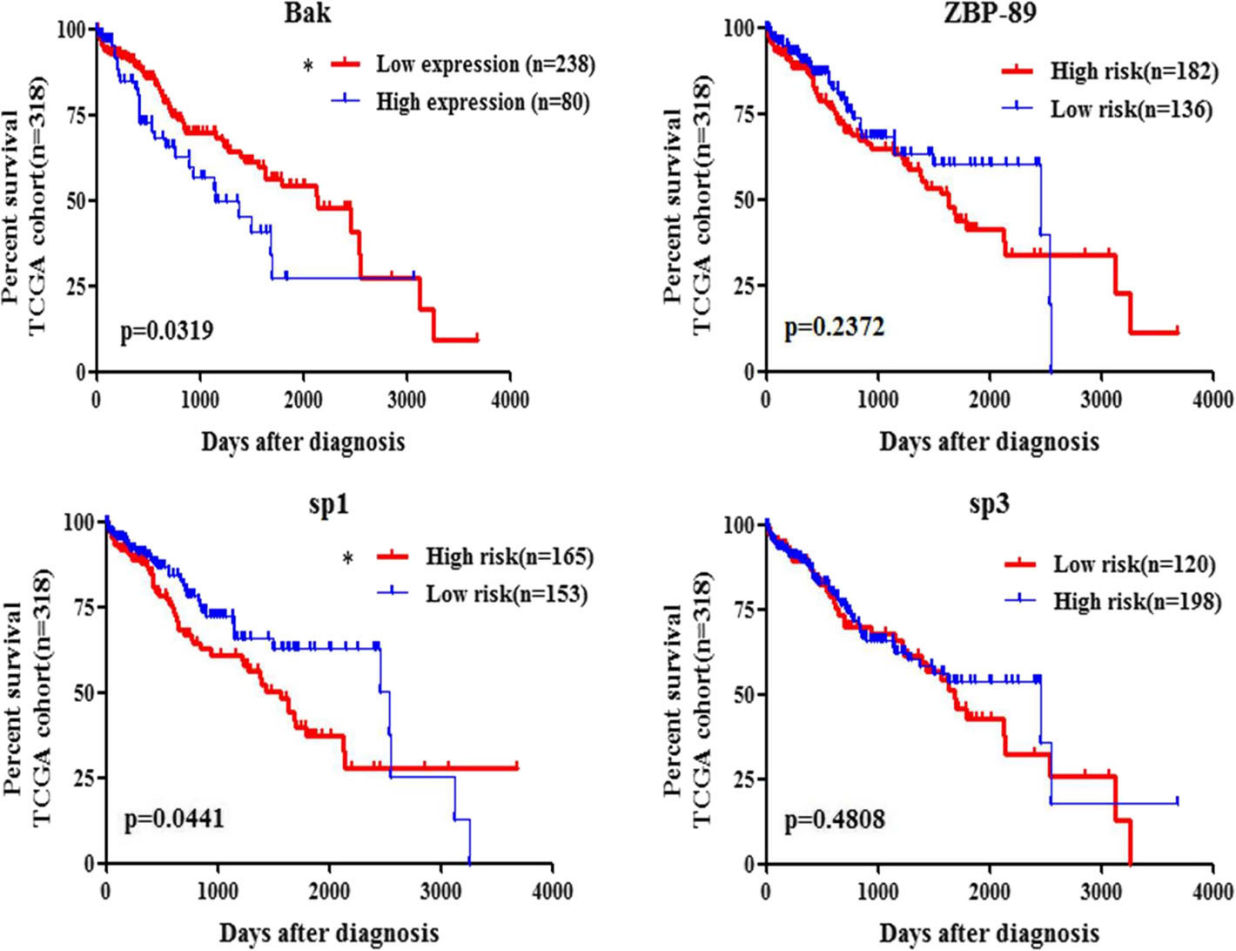
Association of Sp1/Sp3 and ZBP-89 in patient survival. In Kaplan–Meier survival analysis, results showed different Bak or Sp1 expression was associated with patient survival. However, they were reversely correlated. High expression of Bak might predict poorer survival time, whereas high level of Sp1 could benefit patient survival

In order to explore if the expression of Bak, Sp1, Sp3 and ZBP-89 are associated with patients survival, we performed Kaplan–Meier survival analysis. Firstly, ROC curve was used to dichotomize these genes into high expression group and low expression group of all 318 HCC patients from TCGA database. Survival analysis was analyzed using Graphpad Prism 5.0 software, and p < 0.05 was set as statistic significant difference level. Results showed that only Bak and Sp1 expression were significantly correlated with patients survival (Fig. 6, *p* < 0.05, Log-rank test). Results indicated that high expression of Bak was associated with shorter living time. We speculated that high expression Bak might be induced by anti-cancer drug treatment, which damaged hepatocellular function and structure. In the end, patients died of liver dysfunction, multi-organs malfunction et al.

## Discussion

Bak is a major cell death initiator in the apoptotic signaling cascade [25]. It plays a significant role in the therapy and survival of HCC. It has been reported that different agents induce apoptosis in HCC cells by inducing Bak expression, but the mechanism responsible for its regulation in HCC is largely unknown.

Sp1 is a ubiquitous transcription factor and its regulated genes include those involved in proliferation, apoptosis, senescence and angiogenesis. Sp3, which competes with Sp1 for GC-box binding sites, is normally a weak transcriptional activator. However, it could function as a transcriptional activator with similar potency to Sp1 in the absence of acetyltransferases [26]. A similar switch of Sp1 for Sp3 has been observed at the Bak promoter following treatment with the HDAC inhibitors butyrate [18]. However, the relationship between Sp1, Sp3 and Bak is unknown. After the analysis of TCGA HCC database, we observed that Bak transcription in cancer tissue was higher than that in non-cancer tissues, and its transcription was correlated with Sp1 and Sp3 transcription level. No correlation was found between Bak and ZBP-89, neither Sp2 nor Sp4 (data not shown). Further analysis found Bak and Sp1 transcription level were significantly correlated with patients survival. These results illustrated that Bak played an important role in the hepatocarcinogenesis or HCC therapy. In Western blotting analyses, we showed that all selected cell lines (HepG2, Hep3B, PLC/PRF/5,Huh-7, HKCI3, HKCI-4, LO2, and MIHA) were able to constitutively express endogenous Bak. The base level of Bak can be greatly enhanced by MMA (30 nM) treatment and the increase in Bak is functional in terms of apoptotic induction in PLC/PRF/5 cells.

MMA functions as GC-rich DNA sequence binding chemical, acting as a Sp family members’ inhibitor. It was shown to induce apoptosis through increase of Bak expression in colon cancer cells [27]. We observed a regulation of Bak protein level after overexpression or knockdown of Sp1. Therefore we raised the question whether ZBP-89 could compete or cooperate with Sp1 to regulate the expression of the Bak gene. Indeed, Western blotting data showed MMA could not induce Bak expression if without ZBP-89 participation (Fig. 5). Additionally, when cells was treated with ZBP-89 siRNA, the consequence also turned down Bak expression even if Sp1 overexpression (Fig. 5, lane 1, 5th band). Results revealed that Bak expression required ZBP-89 and Sp1 to work simultaneously.

ZBP-89 and Sp family members are members of the zinc finger transcription factor and also subclasses of the C2H2 zinc finger family. ZBP-89 can efficiently inhibit cellular proliferation and induce apoptosis in human cancer cells [28]. In our previous study, we have identified that ZBP-89 might target at − 457 to − 407 region of Bak promoter to induce its expression and subsequently apoptosis [20]. Given the same bifunctional regulatory domains, ZBP-89 and Sp1 can interact to regulate a variety of genes. ZBP-89 appears to interact with Sp1, as binding to an overlapping site in DNA to regulate gastrin, two type I collagen genes [29], and ornithine decarboxylase [30]. In another aspect, many findings also suggested that Sp1 and ZBP-89 regulated target genes in a cooperative manner, like vimentin [12], Pdcd4 [13] and p21waf1/cip1 [31]. In agreement with peers’ finding, we further showed the cooperative function of ZBP-89 and Sp1 on Bak transcription. Besides, the study also revealed their association in patient survival, as well as the Bak expression level.

## Conclusions

Our findings suggested that ZBP-89 and Sp1 overexpression induced Bak expression in a genetic manner. Increased Bak level was associated with poor patient survival, whereas high level of Sp1 is a beneficial factor for patient survival.

## Additional file

Additional file 1 Table S1. Table summary of available patient and chemotherapy information. The supplemental table showed individual HCC patient and treatment one received, and also the therapy duration time since the initial treatment. (XLSX 12 kb)

## Abbreviations

HCC: Hepatocellular carcinoma
MMA: Mithramycin A
Sp1: Sp1 transcription factor
TCGA: The Cancer Genome Atlas
ZBP-89: Zinc binding protein 89
